# Serum lipid profile as a marker of liver impairment in hepatitis B Cirrhosis patients

**DOI:** 10.1186/s12944-017-0437-2

**Published:** 2017-03-01

**Authors:** Sadia Qamar Arain, Farah Naz Talpur, Naseem Aslam Channa, Muhammad Shahbaz Ali, Hassan Imran Afridi

**Affiliations:** 1 National Centre of Excellence in Analytical Chemistry, University of Sindh, Jamshoro, 76080 Pakistan; 2 Institute of Biochemistry University of Sindh, Jamshoro, Pakistan; 3 Chulalongkorn University, Bangkok, Thailand

**Keywords:** Hepatitis B virus, Fatty acids, Cholesterol, GC-FID, Triacylglycerol, High density lipoprotein, Low density lipoprotein, Very low density lipoprotein

## Abstract

**Background:**

Chronic HBV infection is a major cause of Cirrhosis and an important risk factor to develop hepatocellular carcinoma. The study is conducted to find out the changes in the lipid metabolism of HBV-cirrhosis patients.

**Methods:**

In the present study, serum lipid profiles of patients with HBV-cirrhosis were assessed by utilizing micro-lab and gas chromatography, while risk factors for transmission of HBV-cirrhosis studied through the standard questionnaire.

**Results:**

The epidemiological and etiological risk factors strongly associated with HBV-cirrhosis patients compared to controls, included as family history, shave from the barber, blood transfusion (without proper screening), mutual sharing of household contents, positive surgery history, and dental treatment. The HBV-cirrhosis patients have significantly lower level (*p* < 0.001) of lipid profile including total cholesterol (96.65 mg/dl), TAG (82.85 mg/dl), VLDL-C (16.57 mg/dl), LDL-C (68.27 mg/dl), HDL-C (27 mg/dl) and total lipid (424.76 mg/dl) in comparison to controls, indicating hypolipidemia in patients. The MELD score indicated mild prognostic values of the hepatic function for the study group. The result of total fatty acid composition of HBV-cirrhotic patients with comparison of control subjects reveals that palmitic (24.54 g/100 g) and palmitoleic acid (4.65 g/100 g) were significantly (*p* < 0.05) higher whereas eicosatrienoic (0.09 g/100 g), arachidonic (3.57 g/100 g), linoleic (22.75 g/100 g) and α-linolenic acid (0.12 g/100 g) were significantly lower. Marker for stearoyl-CoA desaturase (SCD = ∆9-desaturase) activity i.e. palmitoleic: palmitic (0.2) and oleic: stearic acid (1.5) ratios, originated higher in HBV-cirrhotic patients, while PUFA: SFA (0.6) was lower in HBV-cirrhosis patients as compared with control subjects. The serum SFA and MUFA were increased while PUFA were reduced in both total and free form.

**Conclusion:**

Present study concluded that hypolipidemia observed in HBV-cirrhosis patients, MELD were found to be independent predictors of survival and alteration in fatty acid composition, possibly due to impairment in fatty acid metabolism by enzymatic elongation and desaturation.

**Electronic supplementary material:**

The online version of this article (doi:10.1186/s12944-017-0437-2) contains supplementary material, which is available to authorized users.

## Background

Hepatitis B Virus (HBV) is an enveloped member of the *Hepadnaviridae* family genus *Orthohepadnavirus* [1]. HBV infection with serious long-term morbidity and mortality is one of the most important infectious diseases in the world. More than 2 billion people have been infected with HBV, and 360 million have chronically infected with HBV Worldwide. Approximately 600,000 people died from acute or chronic HBV every year [2]. Chronic HBV infection is a major cause of Cirrhosis along with important risk factor to develop Hepatocellular Carcinoma (HCC) [3].

Viral hepatitis cirrhosis is highly concerned and the major cause of deaths (due to an infectious agent) in Pakistan. Hepatitis B is the major causes of chronic liver diseases, with the prevalence of 3–7%. The cirrhosis developed in 10–20% of chronic Hepatitis B patients within 5–30 years [4].

The formation and clearance of lipoproteins occur in the liver. From the diet and peripheral tissues it receives cholesterol and fatty acids and converts them into lipoprotein complexes, eventually, releases into the blood circulation. The liver diseases disrupt plasma lipids through different ways. The plasma triglyceride and cholesterol reduced in chronic liver disease, due to the lower biosynthetic capacity of lipoprotein [5]. Hepatic impairments are caused by HBV, which in turn relates to the alterations of lipid metabolism [6, 7]. Chronic hepatitis B, C, and cirrhosis of the liver, associated with impaired lipid metabolism reduced total cholesterol and HDL-C in case-control studies. Changes in serum lipids were commonly found in patients with chronic liver disease [8].

The fatty acids play an important role in the pathogenesis of various diseases like metabolic disorders (Diabetes, obesity, and cardiovascular disease) [9, 10]. Steatosis is developed in nonalcoholic fatty liver disease mainly due to altered level of hepatic lipid, particularly a decrease in polyunsaturated fatty acid (PUFA). The gene expression in liver and skeletal muscles are influenced by PUFA, therefore, reduction occurs in fatty acid synthesis, triacylglycerol storage, and fatty acid oxidation is increases. The changes in tissues and PUFA contents moreover affect eicosanoid synthesis, which might further promote the inflammation and steatosis [11].

Free fatty acids (FFAs) are significant mediators of lipotoxicity; act as possible cellular toxins which lead to the lipid over-accumulation. When lipids are over-accumulated in non-adipose tissues, they may enter into non-oxidative deleterious pathways which leads to cell injury and death [12–14]. The elevated level of FFAs in patients with NAFLD correlated with the severity of disease [15].

Several studies have been conducted on dyslipidemia of cirrhotic patients in developed countries, there is a paucity of data in this regard in Pakistan. As there is a high prevalence of cirrhosis in our country, this study conducted to determine the epidemiological and etiological risk factors severity, serum lipid profile, total and free fatty acid composition among patients with HBV-cirrhosis.

## Methods

**Fig. 1:**
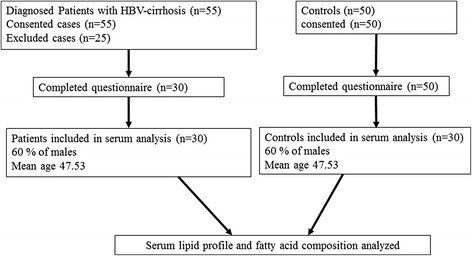
Flow chart of the study

Diagnosed patients of HBV positive cirrhosis admitted to Civil Hospital Hyderabad and Liaquat University of Medical Health Sciences (LUMHS) Hospital Jamshoro were included in this study. All patients were enrolled, have signed a written consent. The risk factors for transmission of HBV-cirrhosis disease were studied by a standard questionnaire, filled by all cases and controls. This study was approved by the ethnic committee, Institute of Biochemistry, University of Sindh, Jamshoro. The flow chart of study is presented in Fig. 1.

### Diagnosis of HBV-cirrhosis patients

The physical examination of patients with HBV cirrhosis, shows specific clinical signs & symptoms as ascites, abdominal wall vascular collaterals, hypertrophic osteoarthropathy, clubbing and asterixis. Constitutional symptoms include weakness, fatigue, anorexia, and weight loss. The ALT is the best screening test for identification of metabolic disorder and drug-induced liver injury, but it has limitation for predicting the degree of inflammation and estimating the severity of fibrosis, the clinical evaluation of the patients strongly favor to liver cirrhosis. The elevated level of Alanine aminotransferase (ALT) was considered as indicators of hepatocellular injury and responsible for the altered lipid profile in HBV cirrhosis patients. Wong et al. [16] reported that patients with abnormal ALT had a similar degree of necroinflammation and fibrosis and their metabolic profile was similar. ALT level largely reflected the degree of hepatic steatosis instead of necroinflammation. No single ALT cutoff could achieve reasonable sensitivity and specificity in predicting the presence of NASH or significant liver fibrosis. The present study includes all patients with positive HBV DNA, analyzed by PCR and having the higher level of ALT. The ultrasonography and liver biopsy were also performed by the hospitals and after interpretation of sample slides microscopically the findings were evidence of regenerative nodules of hepatocytes, surrounded by fibrous connective tissue that bridges between portal tracts, confirm the presence of cirrhosis.

Age and gender matched controls with Hepatitis B negative history (confirmed by non-reactive ELISA) were included in the study.

### Sample collection and analysis

5 ml intravenous blood samples were collected from HBV-positive cirrhosis patients and healthy controls (HCV negative) after 14 h overnight fasting. Serum was separated and stored at −40 °C until analyzed for lipid profile and fatty acids by micro-lab 300 and gas chromatography (GC 8700, Perkin–Elmer Ltd). Lipid profile performed by kit method (Merck, Germany) included total cholesterol (TC), triacylglycerol (TAG), high density lipoprotein–cholesterol (HDL-C), low density lipoprotein–cholesterol (LDL-C), very low density lipoprotein–cholesterol (VLDL-C) and total lipid (TL) [17].

FA’s composition was analyzed as total fatty acid (TFA) and free fatty acid (FFA) as per reported method [18]. Peaks were identified by authentic standards supplied by Fluka Chemika (Buchs, Switzerland). All solvents and reagents used during the study were of analytical grade. FA composition was reported as a relative percentage of the total peak area.

### Exclusion criteria

After taking complete history, laboratory reports (including thyroid profile, fasting blood sugar, serum urea, uric acid, creatinine and complete picture of blood) and the physical examination by the expert physician, patients were excluded from study with malnutrition, hepatitis co-infection, malabsorption, hypertension, diabetic and hyperthyroidism, renal failure, malignancy and immunoglobulin disorders.

### Statistical analysis

The values were expressed as mean ± SD. For the association between the groups (controls vs. patients) student’s *t*-test or the Mann-Whitney *U* test was utilized with SPSS version 15 (SPSS Inc. Chicago, IL). Multivariable logistic regression analysis was performed by using the SAS statistical software (version 9.1; SAS Institute, Inc., Cary, North Carolina) to determine the independent association of each factor with HBV-cirrhosis patients. Odds ratios and 95% confidence intervals (CI) were calculated to estimate the risk factor. Tests for trend were performed by using the means within each category in the logistic-regression model. Quartile cut points were determined by the distribution of the fatty-acid levels among the referents, and the lowest quartile was used as the reference category. The significant variation observed when the *P* value was less than 0.05.

MELD score was calculated by the “Online UNOS MELD calculator”:

$$ \mathrm{M}\mathrm{E}\mathrm{L}\mathrm{D}\kern2.50pt \mathrm{S}\mathrm{c}\mathrm{o}\mathrm{r}\mathrm{e}=0.957\times {\mathrm{Log}}_{\mathrm{e}}\left(\mathrm{creatinine}\left[\mathrm{mg}/\mathrm{dl}\right]\right)+0.378\times {\mathrm{Log}}_{\mathrm{e}}\left(\mathrm{total}\kern2.50pt \mathrm{bilirubin}\left[\mathrm{mg}/\mathrm{dl}\right]\right)+1.120\times {\mathrm{Log}}_{\mathrm{e}}\left(\mathrm{INR}\right)+0.64. $$

## Results

Among 55 patients, 25 patients were excluded due to the consideration of exclusion criteria while remaining thirty patients enrolled in the study. Sixty percent of males included with overall male to female ratio of 1.5: 1. The median age 47.53 years (range, 29–70 years) was also identified for the study group.

The epidemiological risk factors of the HBV-cirrhosis patients and controls were strongly associated in multivariable analysis included as family history and shave from a barber shop. The strong associations were also observed among patients used to chew betel leaf, areca nut and tobacco/Gutka in the dietary habits (Table 1).

**Table 1:** Epidemiological risk factors for HBV-cirrhosis patients and controls

Variables	Cases (n = 30)	Controls(n = 50)	Odds ratio	95%Confidence intervals	*P* value
Education					
• No education	10	11	1.95	0.477–8.149	0.455
• Primary	4	6	1.43	0.234–8.762	0.960
• Matric	6	8	1.61	0.325–8.084	0.752
• Intermediate	3	10	0.64	0.100–3.853	0.869
• Graduation and above	7	15	1.00	(Reference)	
Marital status					
• Married	18	30	1.00	0.360–2.790	1.000
• Unmarried	12	20	1.00	(Reference)	
Family history					
• Positive	20	10	8.00	2.567–25.834	0.0001
• Negative	10	40	1.00	(Reference)	
Unani/homeopathic treatment					
• Yes	10	15	1.17	0.396–3.426	0.951
• No	20	35	1.00	(Reference)	
Self-medication					
• Yes	12	15	1.56	0.543–4.469	0.502
• No	18	35	1.00	(Reference)	
Shaving					
• Self	7	15	1.00	(Reference)	
• Barber Shop	6	5	2.57	0.463–14.975	0.378
• Both self and barber shop	5	8	1.34	0.255–7.078	0.975
Ear/Nose pricking					
• Yes	12	20	1.00	0.358–2.782	1.000
• No	18	30	1.00	(Reference)	
Addiction of					
• Areca nut	10	6	2.19	0.608–8.154	0.287
• Betel leaf with areca nut and tobacco/Gutka	6	4	1.98	0.420–9.715	0.527
• Moist powdered tobacco snuff	2	1	2.64	0.169–78.024	0.842
Smokers	10	11	1.77	0.575–5.488	0.394
Non smokers	20	39	1.00	(Reference)	

The main etiologies of hepatitis B liver cirrhosis are summarized in Table 2. Blood transfusion (without proper screening), mutual sharing of household contents, positive surgery history, and dental treatment were positively associated with the disease.

**Table 2:** Etiological Risk Factors of Hepatitis B cirrhosis patients and controls

Transmission route	Cases (n = 30)	Controls(n = 50)	Odds ratio	95% Confidence intervals	*P* value
Blood transfusion (without proper screening)					
• Yes	11	7	3.56	1.061–12.220	0.038
• No	19	43	1.00	(Reference)	
Mutual sharing of house hold contents					
• Yes	25	42	7.50	2.217–26.946	0.0001
• No	05	08	1.00	(Reference)	
Surgery history					
• Positive	9	5	3.86	1.009–15.374	0.048
• Negative	21	45	1.00	(Reference)	
Dental treatment					
• Yes	12	11	3.87	1.192–12.827	0.021
• No	18	39	1.00	(Reference)	

It was prominent from the lipid profile data of cirrhosis patients with hepatitis B that the confounding factors (smoking, addiction of Betel leaf with areca nut and tobacco/gutka and moist powdered tobacco snuff) were correlated with disease. The strong correlation was seen in the smokers with HDL-C (R^2^ = 0.42) and the weak association were seen with others confounding factors (Table 3).

**Table 3:** Regression analysis of confounding factors for lipid profile in HBV cirrhosis patients

Confounding factors	Coefficient of determination (R^2^)					
	Total Cholesterol	TAG	HDL-C	LDL-C	VLDL-C	Total lipid
Smokers	0.15	0.11	0.42	0.11	0.18	0.26
Addiction of Areca nut	0.13	0.18	0.20	0.17	0.18	0.26
Addiction of Betel leaf with areca nut and tobacco/Gutka	0.01	0.21	0.23	0.10	0.01	0.10
Addiction of Moist powdered tobacco snuff	0.01	0.05	0.05	0.01	0.05	0.10

For the patients with chronic liver disease specifically, cirrhosis the Model for End-Stage Liver Disease (MELD) were applied to estimate the disease severity and survival, hence helpful for clinical professionals as decision-making tools in patient care. Clinical history and demographic data were collected during filling of the questionnaire, the laboratory results like Serum Bilirubin, Serum Creatinine, and INR were recorded. The patients were classed in three categories as per their MELD scores. Although patients had different levels for serum Bilirubin, Serum creatinine, and INR but the laboratory results collected within the groups as per their values. Five patients with serum bilirubin levels were found in between 2.7 mg/dl to 2.1 mg dl, Serum Creatinine level more than 1.9 mg/dl have INR ration from 1.7 to 1.6; collectively their MELD score was 22.3 which predicted 19.6% mortality, further the 12 patients with MELD score 19.2 predicted 6% mortality and remaining 13 patients with MELD score 16 had more than 5% of mortality prediction (Table 4).

**Table 4:** Model for end stage liver disease (MELD)

Number of Patients	Serum Bilirubin mg/dl	Serum Creatinine mg/dl	INR ratio	MELD Score
5	2.7–2.1	>1.9	>1.7–1.6	22.3
12	2.0–1.8	1.8–1.6	1.5	19.2
13	<1.7	<1.5	<1.5	16

The HBV-cirrhosis patients have a significantly lower level (*p* < 0.001) of lipid profile including total cholesterol, TAG, VLDL-C, LDL-C, HDL-C, and total lipid in comparison to controls (Table 5).

**Table 5:** Lipid profile of HBV cirrhosis patients in comparison of controls

Lipid profile (mg/dl)	Controls	Patients
Total cholesterol	171.43 ± 17.7	96.65 ± 22.24^*^
VLDL-C	26.48 ± 5.0	16.57 ± 3.8^*^
LDL-C	110.7 ± 15.7	68.27 ± 17.8^*^
HDL-C	48.23 ± 7.1	27 ± 9.6^*^
Triglyceride	132.4 ± 25.4	82.85 ± 19.0^*^
Total Lipid	612.8 ± 47.9	424.76 ± 63.9^*^

* Values are mean ± standard deviation * Different from HBV-cirrhosis patients with healthy controls, *p* < 0.001 (*t* test)

The result of TFA composition of HBV-cirrhotic patients with comparison of control subjects reveals that palmitic and palmitoleic acids were significantly (*p* < 0.05) higher while eicosatrienoic, arachidonic, linoleic, eicosapentaenoic, α-linolenic acids were significantly lower in HBV-cirrhotic patients in comparison to healthy subjects. The myristic, stearic, arachidic and docosenoic acids were low and lignoceric, myristoleic, docosapentaenoic and docosahexaenoic acids were elevated but not significant. The nervonic and eicosapentaenoic acid was not detected in HBV-cirrhosis patients. Marker for stearoyl-CoA desaturase (SCD = ∆9-desaturase) activity i.e. palmitoleic: palmitic and oleic: stearic acid ratios, was found higher in HBV-cirrhotic patients, While PUFA: SFA was lower in HBV-cirrhosis patients as compared with control subjects (Table 6).

**Table 6:** Total fatty acids of HBV cirrhosis patients in comparison of controls

Fatty Acids (g/100 g)	Controls	Patients
C-14:0	1.00 ± 0.9	0.88 ± 0.7
C-16 : 0	18.01 ± 4.0	24.54 ± 3.4^*^
C-18 : 0	16.00 ± 4.6	15.77 ± 3.4
C-20 : 0	0.78 ± 0.6	0.21 ± 0.3^*^
C-24 : 0	0.02 ± 0.06	0.07 ± 0.2
C-14 : 1	0.17 ± 0.4	0.26 ± 0.4
C-16 : 1	2.03 ± 1.6	4.65 ± 0.7^*^
C-18 : 1	22.18 ± 4.8	23.70 ± 2.9
C-22 : 1	1.75 ± 1.4	1.38 ± 0.9
C-24 : 1	0.18 ± 0.5	ND
C-18 : 2	28.72 ± 3.3	22.75 ± 3.2^*^
C-18 : 3	0.59 ± 0.9	0.12 ± 0.3
C-20 : 3	1.35 ± 0.9	0.09 ± 0.1^*^
C-20 : 4	6.31 ± 1.0	3.57 ± 0.8^*^
C-20 : 5	0.71 ± 0.3	ND^*^
C-22 : 5	0.33 ± 0.6	0.87 ± 0.8
C-22 : 6	0.50 ± 0.6	0.51 ± 0.7
C-16 : 1: C-16 : 0	0.1	0.2
C-18 : 1: C-18 : 0	1.3	1.5
PUFA : SFA	1.5	0.6

Values are mean ± standard deviation,* shows significant difference from HBV-cirrhosis patients with comparison of healthy controls a *p* < 0.05 (*t*-test). myristic acid (C14:0), myristoleic acid (C14:1), palmitic acid (C16:0), palmitoleic acid (C16:1), stearic acid (C18:0), oleic acid (C18:1), linoleic acid (C18:2), α-linolenic acid (C18:3), arachidic acid (C20:0), eicosatrienoic acid (C-20:3), arachidonic acid (C20:4), eicosapentaenoic acid (EPA (C20:5), docosenoic acid (C22:1), docosahexaenoic acid (DHA (C22:6), nervonic acid(C24:1), not detected (ND)

The serum FFA profile of HBV-cirrhotic patient’s showed significantly (*p* > 0.05) higher level of palmitic, palmitoleic, stearic and myristoleic acid. The arachidic, docosenoic, linoleic, arachidonic acids were significantly lower (*p* < 0.05) in HBV-cirrhotic patients with comparison to controls. The oleic, docosapentaenoic and docosahexaenoic acid were higher and lignoceric, α-linolenic and eicosatrienoic lower in patients but not significant (Table 7).

**Table 7:** Free fatty acid profile of HBV cirrhosis patients in comparison of controls

Fatty Acids (g/100 g)	Controls	Patients
C-14:0	1.29 ± 0.7	1.34 ± 0.9
C-16 : 0	19.84 ± 3.2	24.63 ± 5.1^*^
C-18 : 0	14.30 ± 5.6	17.80 ± 6.9
C-20 : 0	0.82 ± 0.5	0.16 ± 0.3^*^
C-24 : 0	0.11 ± 0.1	0.009 ± 0.03
C-14 : 1	0.14 ± 0.2	0.45 ± 0.4^*^
C-16 : 1	2.25 ± 0.8	3.58 ± 1.1^*^
C-18 : 1	22.50 ± 5.1	24.36 ± 3.1
C-22 : 1	1.70 ± 0.8	0.88 ± 0.6^*^
C-24 : 1	ND	ND
C-18 : 2	27.99 ± 3.9	20.48 ± 3.8^*^
C-18 : 3	0.29 ± 0.1	0.07 ± 0.1
C-20 : 3	0.61 ± 0.4	0.18 ± 0.3
C-20 : 4	5.40 ± 2.4	2.55 ± 1.2^*^
C-20 : 5	0.54 ± 0.1	ND^*^
C-22 : 5	0.05 ± 0.1	0.12 ± 0.1
C-22 : 6	0.09 ± 0.2	0.33 ± 0.4^*^

Values are mean ± standard deviation,* shows significant difference from HBV-cirrhosis patients with comparison of healthy controls a *p* < 0.05 (*t*-test). myristic acid (C14:0), myristoleic acid (C14:1), palmitic acid (C16:0), palmitoleic acid (C16:1), stearic acid (C18:0), oleic acid (C18:1), linoleic acid (C18:2), α-linolenic acid (C18:3), arachidic acid (C20:0), eicosatrienoic acid (C-20:3), arachidonic acid (C20:4), eicosapentaenoic acid (EPA (C20:5), docosenoic acid (C22:1), docosahexaenoic acid (DHA (C22:6), nervonic acid(C24:1), not detected (ND)

Odds ratios were calculated (Table 8) for HBV-cirrhosis patients and controls by quartile of serum fatty acids. Present study found an increased risk of HBV-cirrhosis progression associated positively with increasing levels of myristic, palmitic, stearic, palmitoleic, oleic and docosenoic acids. The significant association between serum fatty acids and HBV-cirrhosis was found when we compared the odds ratio for the highest quartile with the lowest one, myristic acid 4.5 (95% CI: 0.2, 167.1; p for trend = 0.46), palmitic acid 2.5 (95% CI: 0.2, 48.4; p for trend = <0.01), stearic acid 6.0 (95% CI: 0.1, 1378; p for trend = 0.34), palmitoleic 5.0 (95% CI: 0.2, 297.8; p for trend = <0.01), oleic acid 4.0 (95% CI: 0.1, 361.0; p for trend = 0.19) and docosenoic acids odds ratio was 4.3 (95% CI: 0.1, 423.2; p for trend = 0.23). On the conflicting PUFA was inversely correlated with HBV-cirrhosis progression odds ratio for the highest quartile with the lowest one linoleic acid 0.6 (95% CI: 0.02, 19.3; p for trend = <0.01), α linolenic acid 0.2 (95% CI: 0.01, 5.6; p for trend = 0.05), arachidonic acid 0.8 (95% CI: 0.03, 17.8; p for trend = <0.01) and eicosatrienoic acid odds ratio was 0.2 (95% CI: 0.005, 3.6; p for trend = 0.02).

**Table 8:** Odd ratios for HBV-cirrhosis patients and controls according to quartile of serum fatty acids

Fatty acids	Odds ratio (95% confidence interval)					*P* value
	1st Quartile	2nd Quartile	3rd Quartile	4th Quartile	5th Quartile	
	Reference					
C-14:0	1.00	1.5 (0.03–82.4)	2.2 (0.2–35.6)	3.0 (0.1–124.7)	4.5 (0.2–167.1)	0.46
C-16 : 0	1.00	2.5 (0.2–48.4)	1.7 (0.1–37.0)	1.7 (0.03–101.0)	2.5 (0.2–48.4)	<0.01
C-18 : 0	1.00	3.0 (0.1–119.0)	4.0 (0.2–176.9)	3.0 (0.03–904.8)	6.0 (0.1–1378)	0.34
C-20 : 0	1.00	1.7 (0.1–26.2)	1.3 (0.02–84.0)	1.3 (0.1–31.2)	1.3 (0.02–84.0)	0.01
C-16 : 1	1.00	3.7 (0.2–101.2)	3.3 (0.2–59.4)	2.5 (0.04–196.7)	5.0 (0.2–297.8)	<0.01
C-18 : 1	1.00	3.7 (0.2–143.1)	3.0 (0.1–141.2)	3.0 (0.03–904.8)	4.0 (0.1–361.0)	0.19
C-22 : 1	1.00	3.0 (0.1–119.0)	4.0 (1.2–176.9)	3.0 (0.03–904.8)	4.3 (0.1–423.2)	0.23
C-18 : 2	1.00	0.5 (0.02–8.8)	0.6 (0.03–13.5)	0.6 (0.01–50.0)	0.6 (0.02–19.3)	<0.01
C-18 : 3	1.00	0.2 (0.005–3.7)	0.2 (0.01–5.6)	0.5 (0.01–25.4)	0.2 (0.01–5.6)	0.05
C-20 : 4	1.00	0.4 (0.01–10.4)	0.4 (0.01–10.4)	0.7 (0.02–19.3)	0.8 (0.03–17.8)	<0.01
C-20 : 3	1.00	0.6 (0.03–9.4)	0.3 (0.01–6.6)	0.6 (0.01–29.6)	0.2 (0.005–3.6)	0.02
C-20 : 5	1.00	1.2 (0.02–62.1)	1.2 (0.02–62.1)	1.8 (0.1–25.9)	1.2 (0.07–19.9)	0.04
C-22 : 6	1.00	0.3 (0.01–4.9)	0.8 (0.1–12.9)	0.8 (0.02–41.6)	0.8 (0.02–41.6)	0.36

**Fig. 2:**
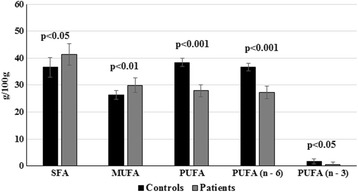
Comparison of serum total SFA, MUFA, PUFA including n–3 and n–6 fatty acids of controls and HBV-cirrhosis patients

The total serum SFA and MUFA were increased significantly (*p* < 0.05) in HBV cirrhotic patients compared with normal controls and PUFA (n–3 and n–6) were lower in HBV-cirrhotic patients in disparity to control subjects (Fig. 2).

**Fig. 3:**
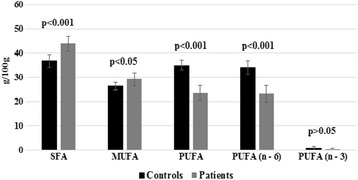
Comparison of free SFA, MUFA, PUFA including n–3 and n–6 fatty acids of controls and HBV-cirrhosis patients

The SFA, MUFA in free form were elevated and PUFA was lower in HBV cirrhotic patients with the contrast of healthy subjects. The Significant variation was seen in FFA including SFA, MUFA, PUFA, and its n–6 form (Fig. 3).

## Discussion

Most of the female patients were married and housewife. The positive family history was significant (p = 0.0001) risk factor associated with HBV-cirrhosis. Khan et al. [19], reported that HBV can be transmitted from house hold contents such as a tooth brush, razors, and nail cutters. Mutual sharing of house hold contents with positive family history arises as a major etiological factor among patients.

Shave from barber shops showed strong association with disease in present study. Janjua et al. [20] in a cross-sectional study of barbers in Rawalpindi/Islamabad in 1999, showed that only 13% of barbers knew that hepatitis could be transmitted by contaminated razors. The current study showed that blood transfusion is strongly related to disease. Approximately 1.5 million units of blood products were transfused every year in Pakistan [21]. Data on the safety of this transfusion process are scanty perhaps due to the lack of a system of reporting infectious or non-infectious adverse events [22]. Luby et al. [23] studied 24 randomly selected blood banks in Karachi in 1995; while 95% had reagents and equipment to test for HBV, only 55% could screen for HIV and 23% for HCV. The positive association of tooth extraction and the role of dental treatment was found as self-determining viral hepatitis risk factor. The persons with lower socioeconomic status exposed to viral hepatitis risk factors due to unhygienic inexpensive dental cares such as tooth extraction which is occasionally accomplished by fake dentists [24].

We have also confirmed the strong association of hepatitis B on hepatocellular carcinoma (HCC) risk by regression analysis of confounding variables. The present study showed the correlation of cigarette smoking with lipid profile. Several theories have been proposed for the role of cigarette smoking in liver carcinogenesis and its potential interaction with viral infection [25, 26]. Cigarette smoke contains several chemicals that are metabolized and activated as carcinogens in the liver and it can, therefore, act as an initiator in the liver carcinogenesis, whereas HBV and HCV mainly act as a promoter through chronic inflammation and cell proliferation through chronic hepatitis and liver cirrhosis. In addition, cigarette smoking may contribute to the progression from chronic HBV and HCV infection to HCC [27]. Chuang et al. [28], reported that smoking seems to interact with both HBV and HCV in determining HCC risk by a pooled analysis of individual subject data, with appropriate adjustment with other risk factors. Shafique et al. [29], reported that raw areca nut users had significantly, reduced HDL-C level. Sajid and Bano [30] reported that deleterious effects of moist powdered tobacco snuff usage caused altered lipid profile and decreased level of HDL-C in the serum of moist powdered tobacco snuff users. Ahmed et al. [31], reported that tobacco chewing has the more harmful effect on lipid profile and lower levels of HDL was found in tobacco chewers.

The MELD score for the patient’s refractory ascites used for assessing the prognosis. However, even though its interpretation can be subjective, ascites was shown to be associated with the poorest prognosis [32]. It was felt that patients with refractory ascites, normal creatinine, and preserved hepatic function could be under-scored with MELD. In particular, it was shown that persistent ascites and low serum sodium identified a subset of patients with relatively low MELD score (below 21) and a high risk of early death [33]. For reference, estimated 3-month mortality, based on the MELD scores was predefined [34]. MELD score 40 or more related to 71.3% mortality, 30–39 for 52.6% mortality, 20–29 for 19.6% mortality, 10–19 for 6.0% mortality and for <9 MELD score 1.9% mortality .

The results of the present study indicated that serum lipids level, total cholesterol, triglycerides HDL-C, LDL-C, VLDL-C and total lipids significantly lower in HBV-cirrhosis patients which indicate hypolipidemia in patients. The measured HDL-C is synthesized in the liver, major injuries to hepatocytes, such as those caused by alcohol consumption, chronic viral hepatitis or cirrhosis of the liver, might produce abnormal liver function and a moderate decrease in levels of total cholesterol and HDL-C [35]. The significantly reduced level of serum total cholesterol and triglycerides found in present study HBV-cirrhosis patients has been confirmed earlier by Ghadir et al. [36] conducted a study on cirrhosis patients in Iran, showed that total cholesterol, HDL, LDL, and triglycerides were all decreased. In patients with cirrhosis, the lower level of cholesterol indicated the severity of liver cell injury, which is associated with impairment of the synthetic ability of the liver. The patients have shown that decreased levels of HDL-C increase the risk of cardiovascular diseases (CVD) [37]. Viral liver infection can be considered a causing factor for atherosclerotic disease progression. The vascular changes seem to start in the liver vasculature in relation to function change rather than to the cause of liver impairment. A reduced nitric oxide production was observed in the hepatic vasculature of both animal and human cirrhotic models. Positive associations among antibody titers, viral hepatitis antigens and severity/mortality from cardiovascular diseases have been supposed, although results are still controversial. Serum concentrations of glucose, total cholesterol and low-density lipoprotein (LDL) cholesterol were significantly lower in anti-HCV-positive [38].

Metabolic changes caused by HBV-cirrhosis are associated with inflammation and fatty liver. In the current study, we establish that the levels of eicosatrienoic, arachidonic, linoleic, eicosapentaenoic, α-linolenic acids were significantly lower in HBV-cirrhotic patients. HBV infection might induce essential FA deficiency in related diseases, as indicated in the HepG2 cell line study [39].

The arachidonic acid was significantly decreased in HBV-cirrhosis patients. Ristic-Medic et al. [40] reported that in alcoholic liver cirrhosis plasma, arachidonic acid deficiency was related to higher mortality rates in patients with advanced liver cirrhosis, and may be an important factor in the pathogenesis that altered renal, immunological and coagulation functions in cirrhosis.

The reduction is observed in the total PUFA level and PUFA/SFA ratio in HBV-cirrhosis patients. Possible reasons include, a poor dietary intake of eicosapentaenoic and linoleic acid and changes in Δ-5, Δ-6 and Δ-9 desaturase enzymes activity due to hepatocellular insufficiency [41] and an increased degradation of PUFA due to lipid peroxidation [42].

The palmitic and palmitoleic acids were significantly high and low stearic was found in HBV-cirrhosis patients. Alvaro et al. [43] found that an impaired FA elongation in cirrhosis may be due to augmented activity of Δ-9 desaturase and decreased the activity of elongase resulted in the decrease level of stearic acid and increased level of palmitoleic acid in cirrhosis patients [44, 45]. The low level of linoleic acid increases the activity of Δ-9 desaturase [46], in addition, increased levels of palmitoleic and oleic acid with the FA status usually characterized by low linoleic acid [47] as observed in malnutrition. High level of palmitic acid in present study HBV-cirrhosis patients does not seem to be affected by malnutrition.

The docosapentaenoic acid increased in current study patients, similarly Puri et al. [48] reported a higher level of docosapentaenoic acid in non-alcoholic fatty liver disease, due to impaired peroxisomal PUFA metabolism.

The palmitic, palmitoleic, stearic and myristoleic acids are elevated in free form in HBV-cirrhosis patients. Most of the literature has focused on the lipotoxic properties of FFAs [49, 50]. Malhi et al. [51] indicated that FFAs can have diverse cellular and metabolic effects particularly stearic acid is more cytotoxic than palmitic acid. Palmitoleic acid reverses the insulin resistance promoted by palmitic acid. The higher level of stearic, palmitic, palmitoleic and myristoleic acid in HBV-cirrhosis patients may represent an adaptive response to fatty acid overload.

The immunomodulating activity is also found in fatty acids [52, 53]. It is the n-3 fatty acids which contained the most potent immunomodulatory activities, and among the n-3 fatty acids, those from fish oil eicosapentaenoic is more biologically potent than α- linolenic acid [54]. The hepatitis B antigen reactivity may be linked with impaired immunity and deficiency of immunomodulating nutrients, it is possible that these agents can alter immunity status causing a decrease in antigen reactivity as observed in current study patients. It is possible that administration of n–3 fatty acid may reduce infection rate and improve liver function recovery in HBV-associated hepatic carcinoma patients after hepatectomy. This improvement is associated with suppressed production of pro-inflammatory cytokines in these patients [55].

## Conclusion

It is concluded that family history, shave from the barber shop, blood transfusion, mutual sharing of household contents, positive surgery history, and dental treatment were the epidemiological and etiological risk factors, strongly associated with HBV-cirrhosis. MELD were found to be independent predictors of survival, with mildly supportive impact for the clinician. Furthermore, hypolipidemia, observed in HBV-cirrhosis patients with an elevated level of SFA and low level of PUFA, possibly due to alteration in fatty acid metabolism by enzymatic elongation and desaturation.

## Additional files

Additional file 1: Serum fatty acid composition and lipid profile data of HBV patients and controls. (XLSX 38 kb)
Additional file 2: Representative GC chromatogram of serum total and free fatty acids of HBV patients and controls along with standards of fatty acid methyl esters.(DOCX 761 kb)

## Abbreviations

CVD: Cardiovascular diseases
FA: Fatty acids
FAS: Fatty-acid synthase
FID: Flame ionization detector
GC: Gas chromatography
HBV: Hepatitis B virus
HDL: High density lipoprotein
LDL: Low density lipoprotein
MUFA: Monounsaturated fatty acid
NAFLD: Nonalcoholic fatty liver disease
PUFA: Poly unsaturated fatty acid
SCD: Stearoyl-CoA desaturase
SFA: Saturated fatty acid
TAG: Triacylglycerol
VLDL: Very low density lipoprotein
