# Supplementary material for: Serum lipid profile as a marker of liver impairment in hepatitis B Cirrhosis patients
Source: Lipids Health Dis. 2017 Mar 1;16:51. doi: 10.1186/s12944-017-0437-2 (PMC5333387; doi:10.1186/s12944-017-0437-2)
Supplement: Additional file 2: — Representative GC chromatogram of serum total and free fatty acids of HBV patients and controls along with standards of fatty acid methyl esters.(DOCX 761 kb) [file 12944_2017_437_MOESM2_ESM.docx]

Additional file 2


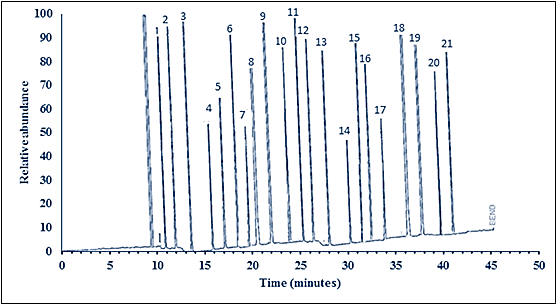


**1**, C- 8:0; **2**,C – 10:0; **3**, C – 12:0; **4**, C – 14:0; **5**, C – 14: 1; **6**, C – 15: 0; **7**, C – 16:0; **8**, C – 16:1; **9**, C – 18:0; **10**, C – 18: 1; **11**, C– 18:2; **12**, C – 20:0; **13**, C – 18:3; **14**, C – 20:3; **15**, C – 22:1; **16**, C – 20:4; **17**, C – 20:5; **18**, C – 22:5; **19**, C – 22:6; **20**, C – 24:0; **21**, C – 24:1.

Figure 1. GC-FID Chromatogram of fatty acids standards


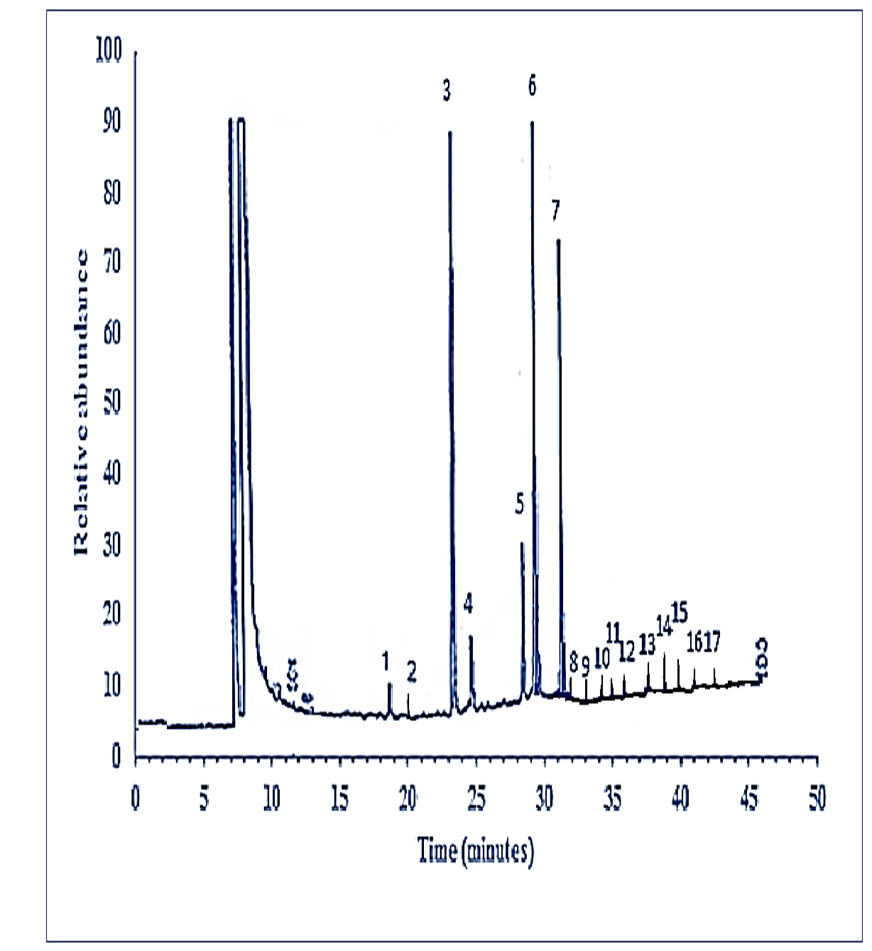


**1,** C – 14:0; **2,** C – 14: 1; **3,** C – 16:0; **4,** C – 16:1; **5,** C – 18:0; **6,** C – 18: 1; **7,** C– 18:2, **8,** C – 20:0; **9,** C – 18:3; **10,** C – 20:3; **11,** C – 22:1; **12,** C – 20:4; **13,** C – 20:5; **14,** C – 22:5; **15,** C – 22:6; **16,** C – 24:0; **17,** C – 24:1.

Figure 2. (a) GC-FID Chromatogram of serum total fatty acids in HBV- cirrhosis Patient


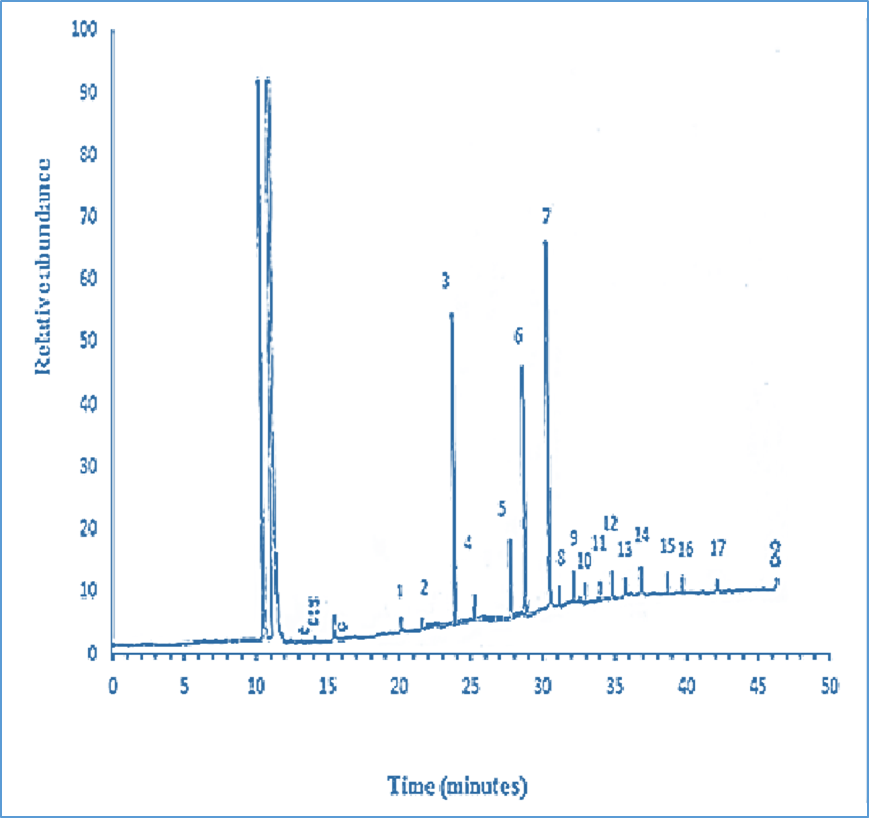


**1,**  C – 14:0; **2,** C – 14: 1; **3,** C – 16:0; **4,** C – 16:1; **5,** C – 18:0; **6,** C – 18: 1; **7,** C– 18:2, **8,** C – 20:0; **9,** C – 18:3; **10,** C – 20:3; **11,** C – 22:1; **12,** C – 20:4; **13,** C – 20:5; **14,** C – 22:5; **15,** C – 22:6; **16,** C – 24:0; **17,** C – 24:1.

Figure 2. (b) GC-FID Chromatogram of serum total fatty acids in control subject


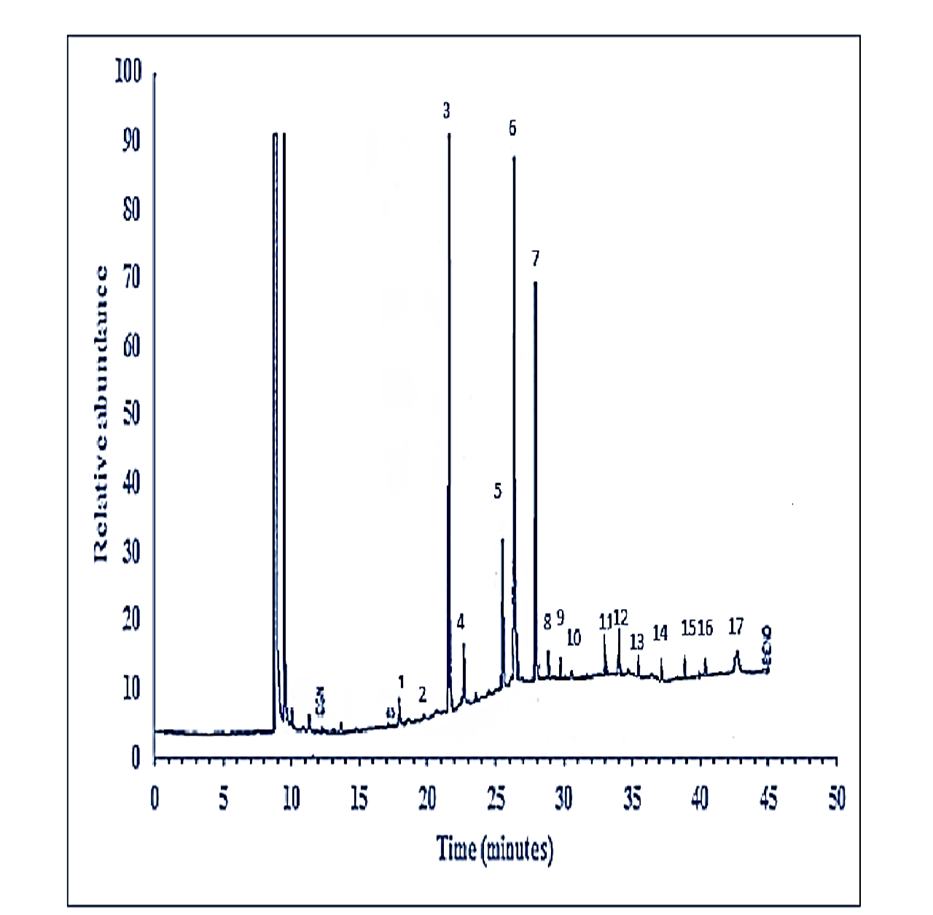


**1**, C – 14:0; **2**, C – 14: 1; **3**, C – 16:0; **4,** C – 16:1; 5,C – 18:0; 6, C – 18: 1; 7, C– 18:2, 8, C – 20:0; 9, C – 18:3; 10, C – 20:3; 11, C – 22:1; 12, C – 20:4; 13, C – 20:5; 14, C – 22:5; 15, C – 22:6; 16, C – 24:0; 17, C – 24:1.

Figure 3. (a) GC-FID Chromatogram of serum free fatty acids in HBV- cirrhosis patient


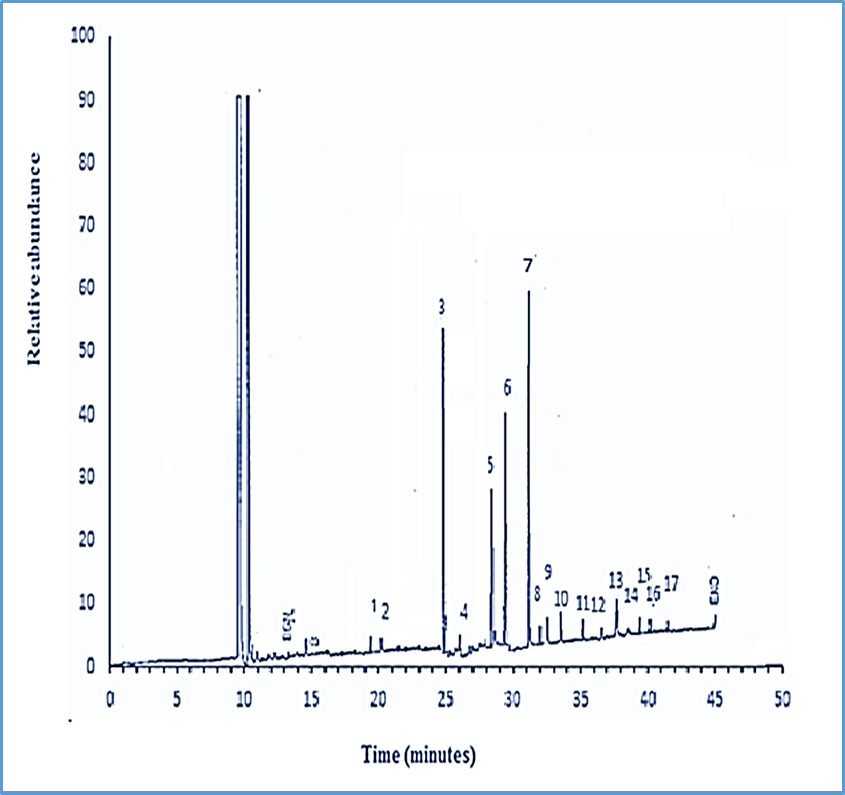


**1,** C – 14:0; **2**, C – 14: 1; **3,** C – 16:0; **4,** C – 16:1; **5,** C – 18:0; **6,** C – 18: 1; **7,** C– 18:2, **8,** C – 20:0; **9,**  C – 18:3; **10,** C – 20:3; **11,** C – 22:1; **12,** C – 20:4; **13,** C – 20:5; **14,** C – 22:5; **15,** C – 22:6; **16,** C – 24:0; **17,** C – 24:1.

Figure 3. (b) GC-FID Chromatogram of serum free fatty acids in control subject
